# Supplementary material for: Immune-mediated genetic pathways resulting in pulmonary function impairment increase lung cancer susceptibility
Source: Nat Commun. 2020 Jan 7;11:27. doi: 10.1038/s41467-019-13855-2 (PMC6946810; doi:10.1038/s41467-019-13855-2)
Supplement: Supplementary file 1 — Supplementary Information [file 41467_2019_13855_MOESM1_ESM.pdf]

Supplementary Table 1: Functional partitioning of array-based heritability for FEV_1, based on genome-wide summary statistics from the UK Biobank cohort and UK Biobank-specific LD scores.

Category	Pr(SNPs)	Pr(h_g)	SE	Enrichment	SE	Enrichment P
H3K4me1 Trynka (500bp window)	0.606	0.892	0.024	1.473	0.039	1.86E-24
SuperEnhancer Hnisz (500bp window)	0.170	0.339	0.014	1.989	0.084	3.35E-24
CpG Content 50kb	0.010	0.012	0.0002	1.201	0.017	7.64E-24
H3K27ac Hnisz (500bp window)	0.420	0.676	0.023	1.609	0.054	8.05E-23
H3K27ac Hnisz	0.389	0.666	0.023	1.712	0.060	1.02E-22
Background Selection Statistic	0.178	0.242	0.006	1.364	0.034	1.91E-20
SuperEnhancer Hnisz	0.167	0.333	0.017	1.992	0.099	1.70E-19
Conserved Primate phastCons (500bp window)	0.176	0.568	0.041	3.230	0.232	3.12E-18
Conserved LindbladToh (500bp window)	0.330	0.617	0.031	1.866	0.095	1.14E-16
Conserved Primate phastCons	0.019	0.251	0.023	13.022	1.207	1.58E-16
H3K9ac Trynka	0.125	0.488	0.041	3.889	0.331	3.41E-16
Conserved Mammal phastCons (500bp window)	0.339	0.674	0.041	1.986	0.121	8.07E-14
H3K27ac PGC2 (500bp window)	0.335	0.567	0.032	1.690	0.095	5.03E-13
H3K9ac Trynka (500bp window)	0.230	0.518	0.040	2.256	0.173	5.44E-13
Intron UCSC (500bp window)	0.397	0.514	0.015	1.296	0.037	2.85E-12
DGF ENCODE (500bp window)	0.538	0.858	0.043	1.593	0.081	1.78E-11
Bivariate Flanking TSS/Enhancer	0.014	0.111	0.015	8.183	1.083	1.33E-10
Conserved LindbladToh	0.026	0.306	0.040	11.923	1.575	1.98E-10
H3K4me1 Trynka	0.424	0.760	0.053	1.793	0.125	5.71E-10
Conserved Vertebrate phastCons	0.029	0.261	0.035	8.861	1.181	7.37E-10
H3K4me3 Trynka (500bp window)	0.255	0.524	0.043	2.053	0.169	1.16E-09
Enhancer Hoffman (500bp window)	0.090	0.238	0.025	2.644	0.276	2.57E-09
Conserved Mammal phastCons	0.021	0.234	0.034	10.893	1.596	4.66E-09
H3K4me3 Trynka	0.133	0.341	0.035	2.561	0.260	5.42E-09
H3K27ac PGC2	0.269	0.520	0.043	1.935	0.160	1.32E-08
TSS Hoffman (500bp window)	0.034	0.125	0.016	3.629	0.458	1.58E-08
DHS Trynka (500bp window)	0.496	0.754	0.045	1.521	0.091	5.49E-08
Conserved Vertebrate phastCons (500bp window)	0.407	0.655	0.044	1.610	0.107	6.69E-08
TFBS ENCODE (500bp window)	0.341	0.587	0.046	1.720	0.136	2.97E-07
DGF ENCODE	0.136	0.449	0.057	3.302	0.422	3.70E-07
Fetal DHS Trynka (500bp window)	0.283	0.502	0.046	1.773	0.162	5.83E-06
BLUEPRINT DNA methylation MaxCPP	0.032	0.100	0.014	3.158	0.444	6.00E-06
Bivariate Flanking TSS/Enhancer (500bp window)	0.031	0.118	0.019	3.797	0.625	1.03E-05
TSS Hoffman	0.018	0.094	0.017	5.287	0.967	1.48E-05
Coding UCSC	0.014	0.098	0.018	6.873	1.286	1.63E-05
Intron UCSC	0.387	0.459	0.017	1.185	0.043	2.19E-05
DHS Trynka	0.166	0.438	0.060	2.636	0.363	2.29E-05

DHS peaks Trynka	0.111	0.344	0.057	3.114	0.519	9.96E-05
Non-synonymous	0.003	0.037	0.008	13.481	3.069	1.07E-04
TFBS ENCODE	0.131	0.331	0.051	2.524	0.392	1.10E-04
Enhancer Hoffman	0.042	0.153	0.030	3.649	0.707	2.36E-04
UTR 3 UCSC	0.011	0.078	0.018	7.006	1.613	2.79E-04
H3K4me1 peaks Trynka	0.170	0.374	0.056	2.204	0.329	3.01E-04
UTR 3 UCSC (500bp window)	0.026	0.085	0.017	3.229	0.626	4.75E-04
Coding UCSC (500bp window)	0.064	0.123	0.017	1.934	0.261	5.29E-04
Fetal DHS Trynka	0.084	0.256	0.049	3.051	0.582	6.17E-04
H3K4me3 peaks Trynka	0.042	0.144	0.033	3.468	0.800	1.99E-03
GERP.RSsup4	0.008	0.080	0.024	9.874	2.933	3.07E-03
H3K9ac peaks Trynka	0.038	0.147	0.037	3.824	0.955	3.34E-03
GTEx eQTL MaxCPP	0.010	0.040	0.010	3.889	1.010	5.75E-03
BLUEPRINT H3K27acQTL MaxCPP	0.017	0.048	0.013	2.898	0.815	0.022
BLUEPRINT H3K4me1QTL MaxCPP	0.013	0.035	0.010	2.645	0.768	0.032
UTR 5 UCSC	0.005	0.027	0.010	4.881	1.761	0.033
Promoter UCSC (500bp window)	0.057	0.084	0.017	1.482	0.304	0.113
Promoter UCSC	0.046	0.074	0.019	1.607	0.404	0.132
Enhancer Andersson	0.004	0.019	0.010	4.472	2.365	0.144
Promoter Flanking Hoffman (500bp window)	0.033	0.060	0.019	1.812	0.583	0.163
CTCF Hoffman (500bp window)	0.071	0.099	0.026	1.392	0.368	0.289
Enhancer Andersson (500bp window)	0.019	0.030	0.012	1.566	0.617	0.358
Transcr Hoffman	0.346	0.389	0.047	1.123	0.136	0.369
Synonymous	0.003	0.012	0.011	3.791	3.561	0.433
UTR 5 UCSC (500bp window)	0.027	0.034	0.014	1.275	0.515	0.594
Weak Enhancer Hoffman	0.021	0.018	0.020	0.837	0.975	0.867
CTCF Hoffman	0.024	0.026	0.022	1.110	0.928	0.906
Weak Enhancer Hoffman (500bp window)	0.089	0.089	0.026	1.001	0.296	0.998

Supplementary Table 2: Functional partitioning of array-based heritability for FEV_1/FVC, based on genome-wide summary statistics from the UK Biobank cohort and UK Biobank-specific LD scores.

Category	Pr(SNPs)	Pr(h_g)	SE	Enrichment	SE	Enrichment P
H3K27ac Hnisz	0.389	0.800	0.030	2.055	0.077	4.18E-23
SuperEnhancer Hnisz	0.167	0.386	0.020	2.309	0.119	9.57E-22
SuperEnhancer Hnisz (500bp window)	0.170	0.397	0.019	2.330	0.111	1.31E-21
H3K27ac Hnisz (500bp window)	0.420	0.768	0.031	1.826	0.075	4.48E-21
H3K4me1 Trynka (500bp window)	0.606	0.922	0.032	1.522	0.052	5.38E-19
CpG Content 50kb	0.010	0.012	0.0002	1.187	0.020	3.84E-17
Background Selection Statistic	0.178	0.226	0.006	1.272	0.035	1.51E-15
H3K27ac PGC2 (500bp window)	0.335	0.612	0.035	1.825	0.106	9.04E-15
H3K9ac Trynka	0.125	0.485	0.047	3.871	0.378	5.98E-13
H3K9ac Trynka (500bp window)	0.230	0.607	0.051	2.639	0.221	2.00E-12
Conserved Primate phastCons (500bp window)	0.176	0.547	0.050	3.109	0.282	2.15E-12
H3K4me3 Trynka (500bp window)	0.255	0.616	0.050	2.413	0.195	3.83E-12
Fetal DHS Trynka (500bp window)	0.283	0.655	0.050	2.311	0.178	7.85E-11
Enhancer Hoffman (500bp window)	0.090	0.279	0.030	3.107	0.329	4.02E-10
DGF ENCODE (500bp window)	0.538	0.862	0.049	1.601	0.092	7.56E-10
Conserved Mammal phastCons (500bp window)	0.339	0.702	0.058	2.070	0.171	1.74E-09
H3K4me1 Trynka	0.424	0.800	0.059	1.888	0.139	4.28E-09
H3K4me3 Trynka	0.133	0.364	0.041	2.739	0.306	1.17E-08
Fetal DHS Trynka	0.084	0.420	0.059	5.010	0.698	1.33E-07
Bivariate Flanking TSS/Enhancer	0.014	0.131	0.022	9.685	1.638	2.47E-07
Conserved Vertebrate phastCons (500bp window)	0.407	0.686	0.056	1.686	0.136	5.00E-07
DHS Trynka (500bp window)	0.496	0.777	0.056	1.566	0.114	7.71E-07
Conserved LindbladToh	0.026	0.212	0.038	8.239	1.473	9.52E-07
Enhancer Hoffman	0.042	0.211	0.033	5.014	0.776	1.02E-06
Intron UCSC (500bp window)	0.397	0.500	0.020	1.259	0.051	1.95E-06
Conserved LindbladToh (500bp window)	0.330	0.583	0.056	1.763	0.169	3.49E-06
H3K27ac PGC2	0.269	0.512	0.053	1.905	0.199	4.17E-06
Conserved Primate phastCons	0.019	0.165	0.031	8.548	1.610	4.91E-06
Bivariate Flanking TSS/Enhancer (500bp window)	0.031	0.126	0.022	4.050	0.691	1.17E-05
Conserved Mammal phastCons	0.021	0.151	0.032	7.057	1.480	2.48E-05
H3K4me1 peaks Trynka	0.170	0.473	0.071	2.785	0.416	2.62E-05
DHS peaks Trynka	0.111	0.532	0.097	4.809	0.875	3.06E-05
DHS Trynka	0.166	0.584	0.095	3.515	0.574	3.35E-05
DGF ENCODE	0.136	0.462	0.079	3.398	0.582	9.07E-05
Conserved Vertebrate phastCons	0.029	0.148	0.033	5.028	1.108	2.27E-04
TFBS ENCODE (500bp window)	0.341	0.525	0.050	1.539	0.146	2.51E-04
GTEx eQTL MaxCPP	0.010	0.054	0.013	5.239	1.216	3.94E-04

BLUEPRINT DNA methylation MaxCPP	0.032	0.102	0.019	3.206	0.585	4.10E-04
TSS Hoffman (500bp window)	0.034	0.110	0.022	3.213	0.630	5.89E-04
H3K9ac peaks Trynka	0.038	0.199	0.049	5.168	1.279	8.69E-04
H3K4me3 peaks Trynka	0.042	0.170	0.042	4.093	1.011	1.82E-03
Intron UCSC	0.387	0.465	0.027	1.200	0.069	4.87E-03
Coding UCSC (500bp window)	0.064	0.147	0.030	2.301	0.475	6.36E-03
TFBS ENCODE	0.131	0.311	0.067	2.373	0.514	7.18E-03
UTR 3 UCSC	0.011	0.048	0.014	4.325	1.226	7.51E-03
TSS Hoffman	0.018	0.079	0.024	4.407	1.326	9.21E-03
Coding UCSC	0.014	0.059	0.019	4.125	1.332	0.018
BLUEPRINT H3K4me1QTL MaxCPP	0.013	0.037	0.010	2.759	0.743	0.019
GERP.RSsup4	0.008	0.061	0.025	7.448	3.036	0.031
Enhancer Andersson	0.004	0.031	0.013	7.128	2.967	0.043
UTR 3 UCSC (500bp window)	0.026	0.066	0.020	2.482	0.766	0.052
BLUEPRINT H3K27acQTL MaxCPP	0.017	0.045	0.015	2.735	0.888	0.053
Non-synonymous	0.003	0.016	0.008	5.847	3.040	0.117
UTR 5 UCSC (500bp window)	0.027	0.045	0.017	1.669	0.619	0.274
Transcr Hoffman	0.346	0.408	0.058	1.179	0.168	0.293
WeakEnhancer Hoffman (500bp window)	0.089	0.120	0.036	1.352	0.403	0.379
WeakEnhancer Hoffman	0.021	0.041	0.024	1.960	1.145	0.398
Transcr Hoffman (500bp window)	0.762	0.729	0.041	0.956	0.054	0.419
CTCF Hoffman	0.024	0.005	0.028	0.206	1.192	0.504
PromoterFlanking Hoffman (500bp window)	0.033	0.049	0.024	1.467	0.718	0.514
Promoter UCSC (500bp window)	0.057	0.068	0.022	1.192	0.381	0.614
CTCF Hoffman (500bp window)	0.071	0.058	0.031	0.812	0.443	0.670
Enhancer Andersson (500bp window)	0.019	0.026	0.019	1.342	1.006	0.733
UTR 5 UCSC	0.005	0.009	0.014	1.558	2.638	0.832
Synonymous	0.003	0.001	0.014	0.457	4.364	0.901
Promoter UCSC	0.046	0.048	0.026	1.035	0.550	0.950

Supplementary Table 3: Functional partitioning of array-based heritability for FVC, based on genome-wide summary statistics from the UK Biobank cohort and UK Biobank-specific LD scores.

Category	Pr(SNPs)	Pr(h2)	SE	Enrichment	SE	Enrichment P
CpG Content 50kb	0.010	0.012	0.0002	1.230	0.019	2.34E-23
Background Selection Statistic	0.178	0.251	0.006	1.411	0.036	6.87E-23
SuperEnhancer Hnisz (500bp window)	0.170	0.347	0.016	2.037	0.095	5.12E-20
H3K4me1 Trynka (500bp window)	0.606	0.893	0.027	1.474	0.045	9.09E-20
Conserved Primate phastCons (500bp window)	0.176	0.585	0.042	3.325	0.236	2.08E-18
H3K27ac Hnisz	0.389	0.652	0.027	1.675	0.069	2.80E-18
H3K27ac Hnisz (500bp window)	0.420	0.666	0.026	1.583	0.062	9.25E-18
SuperEnhancer Hnisz	0.167	0.344	0.018	2.057	0.109	7.54E-17
Conserved Primate phastCons	0.019	0.253	0.025	13.125	1.290	1.91E-15
H3K9ac Trynka (500bp window)	0.230	0.575	0.040	2.503	0.175	2.37E-15
Conserved LindbladToh (500bp window)	0.330	0.648	0.036	1.961	0.110	6.60E-15
H3K9ac Trynka	0.125	0.481	0.046	3.839	0.367	6.48E-13
Intron UCSC (500bp window)	0.397	0.527	0.016	1.328	0.040	1.29E-12
Conserved Mammal phastCons (500bp window)	0.339	0.706	0.048	2.082	0.140	1.51E-12
H3K27ac PGC2 (500bp window)	0.335	0.585	0.036	1.746	0.107	3.34E-11
DGF ENCODE (500bp window)	0.538	0.874	0.046	1.623	0.085	3.53E-11
Conserved LindbladToh	0.026	0.297	0.039	11.556	1.517	1.51E-10
H3K4me3 Trynka (500bp window)	0.255	0.538	0.043	2.110	0.169	3.76E-10
Conserved Mammal phastCons	0.021	0.242	0.032	11.282	1.506	3.80E-10
H3K4me1 Trynka	0.424	0.769	0.052	1.816	0.123	5.46E-10
Conserved Vertebrate phastCons	0.029	0.254	0.034	8.630	1.148	9.43E-10
H3K4me3 Trynka	0.133	0.350	0.035	2.634	0.266	4.30E-09
Enhancer Hoffman (500bp window)	0.090	0.249	0.026	2.776	0.295	5.72E-09
Bivariate Flanking TSS/Enhancer	0.014	0.132	0.020	9.766	1.447	8.44E-09
DGF ENCODE	0.136	0.506	0.060	3.724	0.443	1.58E-08
DHS Trynka (500bp window)	0.496	0.778	0.047	1.568	0.094	2.63E-08
TSS Hoffman (500bp window)	0.034	0.137	0.019	4.002	0.557	1.51E-07
Conserved Vertebrate phastCons (500bp window)	0.407	0.681	0.050	1.674	0.124	1.67E-07
H3K27ac PGC2	0.269	0.513	0.045	1.909	0.168	2.47E-07
TSS Hoffman	0.018	0.110	0.019	6.152	1.093	4.03E-06
TFBS ENCODE (500bp window)	0.341	0.590	0.052	1.728	0.153	4.54E-06
TFBS ENCODE	0.131	0.392	0.056	2.989	0.428	8.29E-06
DHS Trynka	0.166	0.466	0.064	2.805	0.386	1.14E-05
Bivariate Flanking TSS/Enhancer (500bp window)	0.031	0.132	0.023	4.223	0.729	1.51E-05
DHS peaks Trynka	0.111	0.374	0.061	3.386	0.554	3.68E-05
BLUEPRINT DNA methylation MaxCPP	0.032	0.101	0.016	3.169	0.508	4.61E-05
Coding UCSC	0.014	0.102	0.021	7.153	1.450	5.50E-05

Intron UCSC	0.387	0.461	0.019	1.191	0.048	1.09E-04
H3K4me1 peaks Trynka	0.170	0.379	0.055	2.233	0.321	1.75E-04
Coding UCSC (500bp window)	0.064	0.143	0.021	2.241	0.323	2.21E-04
FetalDHS Trynka (500bp window)	0.283	0.482	0.053	1.702	0.185	2.52E-04
Enhancer Hoffman	0.042	0.160	0.034	3.822	0.804	6.03E-04
UTR 3 UCSC	0.011	0.082	0.020	7.327	1.823	7.48E-04
Non-synonymous	0.003	0.033	0.009	12.121	3.398	1.42E-03
FetalDHS Trynka	0.084	0.243	0.049	2.894	0.587	1.51E-03
UTR 3 UCSC (500bp window)	0.026	0.081	0.018	3.076	0.675	2.41E-03
GERP.RSsup4	0.008	0.077	0.022	9.487	2.753	2.63E-03
GTEx eQTL MaxCPP	0.010	0.045	0.012	4.395	1.140	3.69E-03
BLUEPRINT H3K27acQTL MaxCPP	0.017	0.061	0.015	3.658	0.899	4.00E-03
H3K4me3 peaks Trynka	0.042	0.137	0.036	3.291	0.861	8.58E-03
UTR 5 UCSC	0.005	0.033	0.012	5.952	2.178	0.025
BLUEPRINT H3K4me1QTL MaxCPP	0.013	0.037	0.011	2.767	0.811	0.030
H3K9ac peaks Trynka	0.038	0.125	0.044	3.248	1.131	0.049
PromoterFlanking Hoffman (500bp window)	0.033	0.074	0.022	2.230	0.669	0.068
Promoter UCSC (500bp window)	0.057	0.088	0.019	1.547	0.331	0.099
Promoter UCSC	0.046	0.076	0.020	1.639	0.429	0.137
Synonymous	0.003	0.023	0.014	7.340	4.368	0.150
Transcr Hoffman	0.346	0.419	0.052	1.211	0.150	0.162
Enhancer Andersson (500bp window)	0.019	0.037	0.013	1.966	0.699	0.165
UTR 5 UCSC (500bp window)	0.027	0.046	0.014	1.727	0.536	0.179
Enhancer Andersson	0.004	0.019	0.011	4.303	2.560	0.198
WeakEnhancer Hoffman	0.021	0.037	0.024	1.762	1.127	0.498
CTCF Hoffman	0.024	0.028	0.026	1.173	1.071	0.872
WeakEnhancer Hoffman (500bp window)	0.089	0.093	0.028	1.050	0.311	0.872
CTCF Hoffman (500bp window)	0.071	0.073	0.027	1.026	0.379	0.946

Supplementary Table 4: Partitioned heritability for estimates for lung cancer

Category	Pr(SNPs)	Pr(h2)	SE	Enrichment	SE	Enrichment P
CpG Content 50kb	0.010	0.014	0.001	1.351	0.061	2.07E-07
Background Selection Statistic	0.178	0.251	0.019	1.412	0.108	1.03E-06
SuperEnhancer Hnisz	0.167	0.367	0.048	2.194	0.285	4.40E-06
SuperEnhancer Hnisz (500bp window)	0.170	0.350	0.051	2.052	0.298	7.61E-05
Coding UCSC (500bp window)	0.064	0.364	0.082	5.710	1.295	4.12E-04
Intron UCSC (500bp window)	0.397	0.574	0.055	1.447	0.137	1.11E-03
H3K27ac PGC2	0.269	0.700	0.128	2.605	0.477	1.28E-03
Conserved Vertebrate phastCons (500bp window)	0.407	0.857	0.147	2.106	0.362	3.60E-03
Conserved Mammal phastCons (500bp window)	0.339	0.791	0.149	2.332	0.438	3.93E-03
Conserved Primate phastCons (500bp window)	0.176	0.524	0.130	2.981	0.742	5.34E-03
Conserved LindbladToh (500bp window)	0.330	0.761	0.144	2.302	0.436	5.36E-03
Conserved LindbladToh	0.026	0.322	0.110	12.552	4.268	0.011
H3K27ac Hnisz (500bp window)	0.420	0.670	0.095	1.594	0.227	0.012
DHS Trynka (500bp window)	0.496	0.901	0.157	1.817	0.316	0.013
Conserved Primate phastCons	0.019	0.228	0.086	11.826	4.454	0.013
UTR 3 UCSC (500bp window)	0.026	0.144	0.051	5.452	1.912	0.015
TFBS ENCODE (500bp window)	0.341	0.736	0.163	2.156	0.479	0.018
Conserved Mammal phastCons	0.021	0.447	0.172	20.855	8.021	0.022
Intron UCSC	0.387	0.506	0.062	1.307	0.160	0.041
H3K9ac Trynka (500bp window)	0.230	0.572	0.163	2.489	0.711	0.046
Conserved Vertebrate phastCons	0.029	0.400	0.174	13.592	5.905	0.046
TSS Hoffman (500bp window)	0.034	0.187	0.076	5.451	2.201	0.050
H3K27ac Hnisz	0.389	0.536	0.073	1.379	0.188	0.055
DGF ENCODE (500bp window)	0.538	0.813	0.140	1.510	0.259	0.058
TFBS ENCODE	0.131	0.489	0.188	3.728	1.430	0.064
H3K4me1 Trynka	0.424	0.705	0.158	1.664	0.372	0.084
UTR 5 UCSC (500bp window)	0.027	0.166	0.081	6.185	3.006	0.091
H3K4me1 Trynka (500bp window)	0.606	0.772	0.098	1.274	0.161	0.106
Enhancer Hoffman (500bp window)	0.090	0.225	0.086	2.502	0.955	0.114
Fetal DHS Trynka (500bp window)	0.283	0.557	0.172	1.967	0.606	0.121
Transcr Hoffman	0.346	0.571	0.159	1.651	0.460	0.125
H3K27ac PGC2 (500bp window)	0.335	0.507	0.119	1.512	0.356	0.126
Promoter UCSC (500bp window)	0.057	0.170	0.076	2.984	1.342	0.149
BLUEPRINT DNA methylation MaxCPP	0.032	0.137	0.072	4.306	2.271	0.150
H3K4me3 Trynka (500bp window)	0.255	0.441	0.135	1.729	0.530	0.175
H3K4me3 Trynka	0.133	0.293	0.120	2.204	0.901	0.181
Synonymous	0.003	0.068	0.049	21.735	15.804	0.190
BLUEPRINT H3K27acQTL MaxCPP	0.017	0.071	0.043	4.315	2.573	0.201

GTEx eQTL MaxCPP	0.010	0.046	0.030	4.483	2.908	0.214
Bivariate Flanking TSS/Enhancer	0.014	0.096	0.070	7.050	5.164	0.243
H3K9ac Trynka	0.125	0.261	0.123	2.079	0.983	0.275
GERP.RSsup4	0.008	0.068	0.056	8.287	6.909	0.299
PromoterFlanking Hoffman	0.008	0.070	0.060	8.483	7.258	0.306
BLUEPRINT H3K4me1QTL MaxCPP	0.013	0.047	0.034	3.482	2.515	0.320
H3K4me3 peaks Trynka	0.042	0.163	0.127	3.902	3.049	0.348
Coding UCSC	0.014	0.069	0.060	4.839	4.228	0.367
Promoter UCSC	0.046	0.154	0.124	3.329	2.680	0.387
Enhancer Hoffman	0.042	0.134	0.105	3.182	2.506	0.389
WeakEnhancer Hoffman (500bp window)	0.089	0.202	0.130	2.273	1.461	0.392
Bivariate Flanking TSS/Enhancer (500bp window)	0.031	0.084	0.065	2.682	2.091	0.416
Enhancer Andersson (500bp window)	0.019	0.055	0.057	2.882	2.991	0.534
UTR 3 UCSC	0.011	0.035	0.038	3.106	3.357	0.534
TSS Hoffman	0.018	0.058	0.071	3.267	4.006	0.573
DGF ENCODE	0.136	0.014	0.233	0.100	1.713	0.599
PromoterFlanking Hoffman (500bp window)	0.033	0.068	0.070	2.055	2.122	0.620
FetalDHS Trynka	0.084	0.004	0.172	0.042	2.049	0.633
DHS Trynka	0.166	0.074	0.223	0.445	1.342	0.669
CTCF Hoffman (500bp window)	0.071	0.143	0.179	2.017	2.522	0.681
H3K4me1 peaks Trynka	0.170	0.242	0.214	1.427	1.257	0.733
H3K9ac peaks Trynka	0.038	0.002	0.119	0.047	3.085	0.754
Transcr Hoffman (500bp window)	0.762	0.750	0.099	0.984	0.130	0.900

Supplementary Table 5: Additional 73 independent variants associated with pulmonary function identified and replicated in the UK Biobank cohort and used as genetic instruments in Mendelian randomization analyses of lung cancer risk

CHR	Position (GRCh37)	SNP	Alleles Effect/Other	EAF	Discovery Beta	Discovery SE	Discovery P-value	Replication P-value	Lead Phenotype[1]	INFO	Nearest Gene and Function	
6	7146350	rs4960289	A/G	0.427	-0.0017	0.0002	9.6E-20	3.7E-14	FEV_1/FVC	0.986	*RREB1*	intronic
22	30598516	rs6006399	T/G	0.884	-0.0245	0.0028	1.9E-18	6.3E-05	FEV1	1	*HORMAD2*	intronic
2	36724282	rs1179500	A/C	0.721	-0.0017	0.0002	3.6E-17	6.8E-05	FEV_1/FVC	0.988	*CRIM1*	intronic
16	67560613	rs7196853	T/C	0.897	-0.0025	0.0003	1.3E-16	1.4E-05	FEV_1/FVC	0.994	*FAM65A*	intronic
4	146087979	rs116125427	G/A	0.922	0.0276	0.0034	2.3E-16	1.7E-06	FEV_1	0.987	*OTUD4*	intronic
10	124230612	rs12571363	C/A	0.893	0.0228	0.0028	3.2E-16	1.3E-06	FVC	0.995	*HTRA1*	intronic
8	103131300	rs659398	T/C	0.269	-0.0017	0.0002	4.9E-16	6.4E-05	FEV_1/FVC	0.983	*NCALD*	intronic
16	58062081	rs41418849	T/G	0.940	-0.0031	0.0004	5.2E-16	4.1E-06	FEV_1/FVC	0.949	*MMP15*	intronic
22	30254175	rs9614084	C/T	0.521	-0.0015	0.0002	7.2E-16	4.9E-11	FEV_1/FVC	0.996	*ASCC2*	intergenic
5	157022475	rs72811372	G/A	0.895	-0.0023	0.0003	2.3E-15	8.3E-06	FEV_1/FVC	0.998	*AC008694.2*	intergenic
1	221473248	rs11118683	C/T	0.579	-0.0140	0.0018	2.5E-15	2.7E-06	FVC	0.975	*RP11-421L10.1*	intergenic
14	54410919	rs4444235	T/C	0.536	0.0014	0.0002	8.9E-15	4.4E-06	FEV_1/FVC	1	*MIR5580*	intergenic
5	120078424	rs1125578	A/C	0.461	-0.0014	0.0002	1.1E-14	1.3E-04	FEV_1/FVC	0.991	*RNU4-69P*	intergenic
19	46309203	rs1548029	A/C	0.644	-0.0015	0.0002	1.3E-14	5.5E-11	FEV_1/FVC	0.998	*RSPH6A*	intronic
2	42433247	rs12466981	C/T	0.728	-0.0015	0.0002	2.7E-14	5.9E-06	FEV_1/FVC	0.996	*EML4*	intronic
1	3425867	rs2794359	C/A	0.895	-0.0023	0.0003	2.8E-14	2.0E-10	FEV_1/FVC	0.986	*MEGF6*	intronic
5	158368797	rs7443323	T/A	0.748	0.0151	0.0020	4.6E-14	1.6E-05	FVC	0.992	*EBF1*	intronic
5	43484257	rs79904209	C/T	0.906	0.0229	0.0030	5.6E-14	2.2E-05	FEV_1	1	*TMEM267*	5' UTR
2	178210268	rs6740092	T/A	0.145	-0.0181	0.0025	1.9E-13	4.3E-05	FVC	0.992	*LOC100130691*	ncRNA_intronic
6	32652486	rs9275156	T/C	0.766	0.0016	0.0002	2.4E-13	1.0E-05	FEV_1/FVC	0.998	*HLA-DQB1*	intergenic
18	20032854	rs4800410	A/C	0.596	-0.0134	0.0018	2.6E-13	1.3E-04	FEV_1	0.988	*RP11-863N1.4*	intergenic
2	100568631	rs11887136	G/A	0.868	0.0189	0.0026	7.6E-13	8.9E-05	FEV_1	0.996	*AFF3*	intronic
6	152593102	rs6904757	A/G	0.636	0.0129	0.0018	9.4E-13	1.1E-04	FVC	0.982	*SYNE1*	intronic
11	13166565	rs11022690	T/C	0.545	-0.0013	0.0002	1.2E-12	8.2E-06	FEV_1/FVC	0.995	*RP11-413N13.1*	intergenic
2	69407720	rs10173269	T/G	0.539	-0.0013	0.0002	1.3E-12	1.0E-04	FEV_1/FVC	1	*ANTXR1*	intronic

6	33025953	rs6457709	A/G	0.705	0.0014	0.0002	1.4E-12	5.3E-06	FEV_1/FVC	1	*HLA-DPA1*	intergenic
6	26207174	rs9393688	A/T	0.738	-0.0139	0.0020	1.7E-12	2.9E-05	FVC	0.992	*HIST1H4E*	downstream
6	19837774	rs11759102	C/T	0.840	-0.0171	0.0024	2.3E-12	5.2E-05	FEV_1	0.989	*ID4*	5' UTR
6	22072425	rs196025	A/G	0.368	-0.0130	0.0019	2.4E-12	9.0E-06	FEV_1	0.985	*CASC15*	ncRNA_intronic
8	69570332	rs17387279	T/G	0.819	0.0156	0.0023	5.0E-12	1.8E-04	FVC	0.990	*C8orf34*	intronic
1	92164100	rs4233430	C/T	0.814	-0.0016	0.0002	9.9E-12	1.4E-08	FEV_1/FVC	0.992	*TGFBR3*	intronic
15	77354847	rs4886509	C/A	0.329	-0.0013	0.0002	1.3E-11	1.0E-06	FEV_1/FVC	0.992	*TSPAN3*	intronic
17	60720058	rs12452590	T/G	0.638	0.0013	0.0002	1.5E-11	5.8E-08	FEV_1/FVC	0.974	*MRC2*	intronic
5	170909410	rs11745375	C/T	0.527	-0.0012	0.0002	1.6E-11	1.3E-04	FEV_1/FVC	1	*FGF18*	intergenic
7	19050020	rs28719767	G/C	0.711	-0.0130	0.0020	5.1E-11	7.0E-05	FEV_1	0.983	*HDAC9*	intergenic
22	18357509	rs72490631	T/C	0.773	0.0140	0.0021	5.5E-11	2.0E-06	FEV_1	0.994	*MICAL3*	intronic
2	230258493	rs207672	T/G	0.340	0.0012	0.0002	7.2E-11	1.3E-07	FEV_1/FVC	0.992	*DNER*	intronic
11	12660894	rs7927422	T/C	0.593	0.0115	0.0018	7.5E-11	2.1E-05	FVC	0.995	*TEAD1*	intergenic
3	73554922	rs4677294	T/A	0.641	0.0121	0.0019	9.3E-11	1.6E-04	FEV_1	0.989	*PDZRN3*	intronic
12	65962636	rs10878300	T/G	0.198	-0.0140	0.0022	1.3E-10	8.5E-05	FVC	0.992	*LOC100507065*	ncRNA_intronic
17	38489170	rs2715554	A/G	0.849	0.0016	0.0003	1.5E-10	4.4E-05	FEV_1/FVC	1	*RARA*	intronic
16	88807608	rs750739	A/G	0.833	0.0153	0.0024	1.8E-10	1.0E-04	FEV_1	0.993	*PIEZO1*	intronic
1	92031492	rs2125126	G/A	0.850	-0.0016	0.0003	2.1E-10	8.3E-07	FEV_1/FVC	0.983	*RP11-47K11.2*	intergenic
5	132375622	rs113638840	A/G	0.744	0.0130	0.0020	2.5E-10	2.0E-04	FEV_1	0.996	*HSPA4*	intergenic
14	93507197	rs1956028	T/C	0.874	0.0168	0.0027	4.3E-10	3.4E-07	FEV_1	0.990	*ITPK1*	intronic
1	9498113	rs9660890	T/C	0.803	-0.0014	0.0002	5.8E-10	1.2E-04	FEV_1/FVC	0.992	*RNA5SP40*	upstream
6	90948093	rs58453446	G/C	0.652	-0.0015	0.0002	8.9E-10	3.5E-05	FEV_1/FVC nvsmk	0.998	*BACH2*	intronic
2	169479763	rs10184235	A/G	0.753	-0.0013	0.0002	1.1E-09	1.1E-06	FEV_1/FVC	0.999	*CERS6*	intronic
5	173303392	rs55993676	G/T	0.713	-0.0012	0.0002	1.4E-09	7.4E-05	FEV_1/FVC	0.998	*CPEB4*	intergenic
4	174582067	rs10005540	C/T	0.386	-0.0107	0.0018	2.6E-09	1.7E-04	FVC	0.978	*RANP6*	intergenic
1	22653424	rs4233284	C/G	0.672	0.0109	0.0018	3.2E-09	1.3E-04	FVC	0.993	*RP11-415K20.1*	intergenic
12	28840892	rs12313454	A/G	0.882	-0.0158	0.0027	3.5E-09	9.2E-05	FVC	0.996	*CCDC91*	intergenic
8	64806567	rs1425794	G/T	0.565	-0.0103	0.0017	4.2E-09	1.5E-04	FVC	0.995	*RP11-32K4.1*	ncRNA_intronic

7	140560023	rs13227429	T/C	0.436	0.0102	0.0017	5.6E-09	6.6E-05	FVC	0.994	*BRAF*	intronic
2	67052289	rs17032590	A/G	0.791	-0.0013	0.0002	8.1E-09	8.0E-06	FEV_1/FVC	0.996	*AC009474.1*	intergenic
8	13194983	rs1528624	T/G	0.505	0.0010	0.0002	8.4E-09	7.6E-09	FEV_1/FVC	0.990	*DLC1*	intronic
10	63839417	rs4948502	T/C	0.573	0.0101	0.0018	8.9E-09	3.0E-07	FVC	0.995	*ARID5B*	intronic
5	148203236	rs35684381	T/C	0.854	-0.0015	0.0003	8.9E-09	3.4E-06	FEV_1/FVC	0.979	*ADRB2*	intergenic
4	145557467	rs35797611	T/C	0.968	-0.0029	0.0005	9.6E-09	1.9E-05	FEV_1/FVC	1	*HHIP-AS1*	intergenic
20	3660789	rs603112	T/C	0.157	-0.0014	0.0002	1.2E-08	1.3E-04	FEV_1/FVC	1	*ADAM33*	intronic
3	11642114	rs1561073	T/A	0.262	-0.0012	0.0002	1.6E-08	2.2E-05	FEV_1/FVC	0.992	*VGLL4*	intronic
15	67464291	rs72743477	A/G	0.783	0.0012	0.0002	1.7E-08	1.1E-04	FEV_1/FVC	0.985	*SMAD3*	intronic
7	134567104	rs28517513	G/T	0.751	0.0012	0.0002	2.1E-08	5.7E-05	FEV_1/FVC	0.990	*CALD1*	intronic
11	65324276	rs11227223	C/T	0.945	-0.0022	0.0004	2.2E-08	3.4E-07	FEV_1/FVC	0.995	*LTBP3*	intronic
17	13416372	rs1978218	G/T	0.404	-0.0132	0.0024	2.3E-08	1.4E-04	FVC-nvsmk	0.986	*HS3ST3A1*	intronic
4	90036240	rs17821105	A/T	0.830	0.0013	0.0002	2.5E-08	2.3E-06	FEV_1/FVC	0.997	*TIGD2*	downstream
16	31030344	rs4889526	C/A	0.625	0.0102	0.0018	2.9E-08	9.5E-09	FEV_1	0.998	*STX1B*	intergenic
3	53672471	rs9819463	T/C	0.795	0.0123	0.0022	2.9E-08	2.5E-07	FEV_1	0.994	*CACNA1D*	intronic
9	129416317	rs10987386	C/T	0.813	0.0013	0.0002	3.3E-08	1.2E-08	FEV_1/FVC	0.982	*LMX1B*	intronic
2	43762112	rs77972916	G/A	0.924	-0.0019	0.0003	3.3E-08	3.0E-05	FEV_1/FVC	0.995	*THADA*	intronic
18	38023468	rs4636990	G/A	0.457	-0.0096	0.0017	3.8E-08	7.3E-05	FVC	0.990	*RNU7-145P*	intergenic
8	109446828	rs10089406	T/C	0.404	0.0010	0.0002	4.4E-08	5.0E-06	FEV_1/FVC	0.987	*EIF3E*	intergenic

1. Lead phenotype refers to the lung function trait for which this variant was selected to be a genetic instrument. For each SNP the lead phenotype has the smallest p-value.

Supplementary Table 6: Descriptive characteristics of the OncoArray lung cancer population

		Lung Cancer Cases		Controls	
		Number	(%)	Number	(%)
Age	≤50 years	3112	(12)	6,032	(12)
	>50 years	23025	(88)	44,075	(88)
Sex	Male	18208	(62)	27,178	(53)
	Female	11059	(38)	24,069	(47)
Smoking status	Never smokers	2355	(9)	7,504	(31)
	Ever smokers	23223	(91)	16,964	(69)
	Former smokers	9037	(35)	8,554	(35)
	Current smokers	13356	(52)	7,477	(31)
Histology	Adenocarcinoma	11273	(39)		
	Squamous cell carcinoma	7426	(35)		
	Small cell carcinoma	2664	(9)		
Total		29266	(100)	56,450	(100)

Supplementary Table 7: Mendelian randomization (MR) odds ratio (OR) estimates for lung cancer overall and by histology for a genetically predicted 1-SD decrease in a standardized FEV_1 z-score

Outcome	MR Estimator	N_{SNPs}	OR	(95% CI)	P
Lung Cancer (cases: n=29,266 controls: n=56,450)	IVW-RE	193	1.27	(1.01 – 1.60)	0.041
	Maximum likelihood	193	1.28	(1.12 – 1.47)	3.4×10^{-4}
	Weighted median	193	1.06	(0.86 – 1.32)	0.572
	MR RAPS	193	1.13	(0.91 – 1.40)	0.255
	MR Egger slope	193	1.65	(0.70 – 3.87)	0.251
	Outliers filtered:				
	IVW-RE	157	1.12	(0.97 – 1.30)	0.132
	Maximum likelihood	157	1.12	(0.97 – 1.30)	0.132
	Weighted median	157	1.07	(0.86 – 1.32)	0.554
	MR RAPS	157	1.09	(0.93 – 1.28)	0.299
	MR Egger slope	157	0.84	(0.47 – 1.50)	0.555
Adenocarcinoma (cases: n=11,273 controls: n=56,450)	IVW-RE	192	0.99	(0.79 – 1.26)	0.965
	Maximum likelihood	192	0.99	(0.83 – 1.19)	0.956
	Weighted median	192	1.07	(0.81 – 1.43)	0.633
	MR RAPS	192	1.00	(0.79 – 1.27)	0.987
	MR Egger slope	192	0.91	(0.39 – 2.10)	0.827
	Outliers filtered:				
	IVW-RE	169	1.03	(0.86 – 1.24)	0.714
	Maximum likelihood	169	1.03	(0.85 – 1.26)	0.728
	Weighted median	169	1.08	(0.81 – 1.44)	0.612
	MR RAPS	169	1.05	(0.86 – 1.29)	0.634
	MR Egger slope	169	0.80	(0.39 – 1.66)	0.557
Squamous carcinoma (cases: n=7426) controls: n=56,450)	IVW-RE	190	2.03	(1.43 – 2.86)	6.3×10^{-5}
	Maximum likelihood	190	2.04	(1.64 – 2.54)	1.2×10^{-10}
	Weighted median	190	1.54	(1.09 – 2.17)	0.014
	MR RAPS	190	1.81	(1.30 – 2.50)	3.7×10^{-4}
	MR Egger slope	190	5.43	(1.50 – 19.63)	0.011
	Outliers filtered:				
	IVW-RE	156	1.51	(1.21 – 1.88)	2.2×10^{-4}
	Maximum likelihood	156	1.50	(1.19 – 1.90)	6.7×10^{-4}
	Weighted median	156	1.44	(1.02 – 2.04)	0.040
	MR RAPS	156	1.48	(1.16 – 1.88)	1.7×10^{-3}
	MR Egger slope	156	2.11	(0.89 – 5.02)	0.092

IVW – RE : Inverse variance weighted – multiplicative random effects
RAPS : Robust adjusted profile score – Huber loss function
PRESSO : Randomization Pleiotropy RESidual Sum and Outlier

Supplementary Table 8: Mendelian randomization (MR) odds ratio (OR) estimates for the risk of lung cancer overall and by histology for a genetically predicted 1-SD decrease in a standardized FVC z-score.

Outcome	MR Estimator	N_{SNPs}	OR	(95% CI)	P
Lung Cancer (cases: n=29,266 controls: n=56,450)	IVW-RE	144	1.16	(0.91 – 1.47)	0.232
	Maximum likelihood	144	1.16	(0.98 – 1.37)	0.078
	Weighted median	144	1.05	(0.81 – 1.35)	0.726
	MR RAPS	144	1.09	(0.87 – 1.36)	0.445
	MR Egger slope	144	0.75	(0.28 – 2.04)	0.576
	Outliers filtered:				
	IVW-RE	124	1.14	(0.97 – 1.34)	0.121
	Maximum likelihood	124	1.14	(0.96 – 1.37)	0.144
	Weighted median	124	1.05	(0.81 – 1.37)	0.699
	MR RAPS	124	1.13	(0.94 – 1.36)	0.206
	MR Egger slope	124	0.45	(0.20 – 1.05)	0.067
Adenocarcinoma (cases: n=11,273 controls: n=56,450)	IVW-RE	143	0.92	(0.68 – 1.24)	0.576
	Maximum likelihood	143	0.92	(0.73 – 1.16)	0.462
	Weighted median	143	1.03	(0.72 – 1.46)	0.878
	MR RAPS	143	0.94	(0.70 – 1.26)	0.689
	MR Egger slope	143	0.64	(0.19 – 2.18)	0.478
	Outliers filtered:				
	IVW-RE	124	0.96	(0.76 – 1.22)	0.744
	Maximum likelihood	124	0.96	(0.75 – 1.23)	0.760
	Weighted median	124	1.04	(0.72 – 1.49)	0.842
	MR RAPS	124	0.98	(0.76 – 1.27)	0.904
	MR Egger slope	124	0.92	(0.29 – 2.91)	0.884
Squamous carcinoma (cases: n=7426) controls: n=56,450)	IVW-RE	140	1.67	(1.13 – 2.46)	0.011
	Maximum likelihood	140	1.68	(1.28 – 2.20)	1.8×10^{-4}
	Weighted median	140	1.19	(0.81 – 1.76)	0.381
	MR RAPS	140	1.46	(1.11 – 1.92)	6.5×10^{-3}
	MR Egger slope	140	0.73	(0.14 – 3.70)	0.700
	Outliers filtered:				
	IVW	122	1.27	(0.96 – 1.66)	0.092
	Maximum likelihood	122	1.27	(0.95 – 1.69)	0.103
	Weighted median	122	1.18	(0.78 – 1.78)	0.429
	MR RAPS	122	1.25	(0.93 – 1.69)	0.139
	MR Egger slope	122	0.43	(0.13 – 1.41)	0.167

IVW – RE : Inverse variance weighted – multiplicative random effects

RAPS : Robust adjusted profile score – Huber loss function

Supplementary Table 9: Mendelian randomization (MR) odds ratio (OR) estimates for the risk of lung cancer overall and by histology for a genetically predicted 10% decrease in FEV_1/FVC.

Outcome	MR Estimator	N_{SNPs}	OR	(95% CI)	P
Lung Cancer (cases: n=29,266 controls: n=56,450)	IVW-RE	264	1.18	(1.01 – 1.38)	0.035
	Maximum likelihood	264	1.18	(1.07 – 1.31)	1.6×10^{-3}
	Weighted median	264	1.10	(0.92 – 1.30)	0.296
	MR RAPS	264	1.11	(0.97 – 1.29)	0.137
	MR Egger slope	264	1.15	(0.77 – 1.71)	0.485
	Outliers filtered:				
	IVW-RE	226	1.10	(0.99 – 1.22)	0.082
	Maximum likelihood	226	1.10	(0.98 – 1.23)	0.100
	Weighted median	226	1.12	(0.93 – 1.34)	0.237
	MR RAPS	226	1.09	(0.97 – 1.23)	0.143
	MR Egger slope	255	1.22	(0.91 – 1.64)	0.184
Adenocarcinoma (cases: n=11,273 controls: n=56,450)	IVW-RE	265	1.11	(0.93 – 1.32)	0.245
	Maximum likelihood	265	1.11	(0.97 – 1.27)	0.145
	Weighted median	265	1.16	(0.91 – 1.46)	0.226
	MR RAPS	265	1.12	(0.94 – 1.33)	0.207
	MR Egger slope	265	1.29	(0.84 – 1.98)	0.240
	Outliers filtered:				
	IVW-RE	239	1.16	(1.01 – 1.34)	0.032
	Maximum likelihood	239	1.17	(1.01 – 1.35)	0.039
	Weighted median	239	1.23	(1.00 – 1.53)	0.054
	MR RAPS	239	1.18	(1.02 – 1.38)	0.030
	MR Egger slope	239	1.43	(1.00 – 2.05)	0.052
Squamous carcinoma (cases: n=7426) controls: n=56,450)	IVW-RE	263	1.30	(1.04 – 1.62)	0.022
	Maximum likelihood	263	1.29	(1.10 – 1.53)	2.4×10^{-3}
	Weighted median	263	1.04	(0.79 – 1.36)	0.775
	MR RAPS	263	1.13	(0.91 – 1.40)	0.255
	MR Egger slope	263	1.25	(0.70 – 2.24)	0.446
	Outliers filtered:				
	IVW-RE	239	1.13	(0.95 – 1.34)	0.164
	Maximum likelihood	239	1.13	(0.95 – 1.34)	0.173
	Weighted median	239	1.04	(0.79 – 1.37)	0.785
	MR RAPS	239	1.09	(0.90 – 1.31)	0.372
	MR Egger slope	239	1.03	(0.65 – 1.62)	0.912

IVW – RE : Inverse variance weighted – multiplicative random effects

RAPS : Robust adjusted profile score – Huber loss function

Supplementary Table 10: Mendelian randomization (MR) odds ratio (OR) estimates for risk of lung cancer in never smokers (2355 cases, 7504 controls) corresponding to a 1-SD decrease in FEV_1 and FVC z-scores and 10% decrease in FEV_1/FVC. Genetic instruments for pulmonary function were developed in a separate GWAS of never smokers in the UK Biobank.

Phenotype	MR Estimator	N_{SNPs}	OR	(95% CI)	P
FEV_1	IVW-RE	76	0.99	(0.55 – 1.80)	0.984
	Maximum likelihood	76	0.99	(0.59 – 1.68)	0.982
	Weighted median	76	0.75	(0.36 – 1.58)	0.450
	MR RAPS	76	1.02	(0.55 – 1.88)	0.958
	MR Egger slope	76	0.87	(0.08 – 9.65)	0.912
	Outliers filtered:				
	IVW-RE	68	1.11	(0.69 – 1.80)	0.669
	Maximum likelihood	68	1.11	(0.64 – 1.93)	0.704
	Weighted median	68	0.81	(0.37 – 1.79)	0.606
	MR RAPS	68	1.13	(0.64 – 2.00)	0.674
	MR Egger slope	68	1.29	(0.15 – 11.07)	0.816
FVC	IVW-RE	57	0.79	(0.37 – 1.66)	0.527
	Maximum likelihood	57	0.78	(0.41 – 1.49)	0.452
	Weighted median	57	0.54	(0.21 – 1.41)	0.211
	MR RAPS	57	0.66	(0.30 – 1.44)	0.296
	MR Egger slope	57	0.58	(0.02 – 18.48)	0.758
	Outliers filtered:				
	IVW-RE	52	0.60	(0.33 – 1.10)	0.101
	Maximum likelihood	52	0.59	(0.30 – 1.17)	0.130
	Weighted median	52	0.52	(0.20 – 1.36)	0.181
	MR RAPS	52	0.57	(0.28 – 1.15)	0.115
	MR Egger slope	52	0.42	(0.02 – 7.83)	0.565
FEV_1/FVC	IVW-RE	112	1.60	(1.05 – 2.45)	0.030
	Maximum likelihood	112	1.61	(1.10 – 2.35)	0.014
	Weighted median	112	1.41	(0.78 – 2.57)	0.254
	MR RAPS	112	1.57	(1.03 – 2.37)	0.035
	MR Egger slope	112	0.86	(0.25 – 2.99)	0.809
	Outliers filtered:				
	IVW-RE	103	1.55	(1.09 – 2.19)	0.014
	Maximum likelihood	103	1.56	(1.05 – 2.30)	0.027
	Weighted median	103	1.47	(0.83 – 2.61)	0.186
	MR RAPS	103	1.54	(1.03 – 2.32)	0.035
	MR Egger slope	103	1.50	(0.47 – 4.83)	0.494

IVW – RE : Inverse variance weighted – multiplicative random effects

RAPS : Robust adjusted profile score – Huber loss function

Supplementary Table 11: Mendelian randomization (MR) odds ratio (OR) estimates for risk of lung cancer in smokers (23223 cases, 16964 controls) corresponding to a 1-SD decrease in FEV_1 and FVC z-scores and a 10% decrease in FEV_1/FVC. Genetic instruments for pulmonary function were not specific to smokers.

Phenotype	MR Estimator	N_{SNPs}	OR	(95% CI)	P
	IVW-RE	188	1.26	(0.98 – 1.63)	0.075
	Maximum likelihood	188	1.26	(1.06 – 1.50)	9.1×10^{-3}
	Weighted median	188	1.12	(0.85 – 1.47)	0.433
	MR RAPS	188	1.16	(0.90 – 1.48)	0.246
	MR Egger slope	188	1.76	(0.69 – 4.51)	0.239
FEV_1	Outliers filtered:				
	IVW-RE	163	1.09	(0.91 – 1.31)	0.363
	Maximum likelihood	163	1.09	(0.90 – 1.31)	0.368
	Weighted median	163	1.06	(0.81 – 1.39)	0.666
	MR RAPS	163	1.05	(0.86 – 1.29)	0.600
	MR Egger slope	163	0.81	(0.41 – 1.61)	0.551
	IVW-RE	139	1.20	(0.91 – 1.58)	0.206
	Maximum likelihood	139	1.20	(0.97 – 1.48)	0.100
	Weighted median	139	1.10	(0.80 – 1.53)	0.546
	MR RAPS	139	1.10	(0.85 – 1.43)	0.472
	MR Egger slope	139	1.05	(0.33 – 3.32)	0.935
FVC	Outliers filtered:				
	IVW-RE	124	1.09	(0.88 – 1.33)	0.434
	Maximum likelihood	124	1.09	(0.87 – 1.36)	0.465
	Weighted median	124	1.07	(0.77 – 1.48)	0.685
	MR RAPS	124	1.08	(0.86 – 1.36)	0.525
	MR Egger slope	124	0.62	(0.24 – 1.61)	0.329
	IVW-RE	258	1.21	(1.01 – 1.46)	0.039
	Maximum likelihood	258	1.21	(1.06 – 1.39)	4.5×10^{-3}
	Weighted median	258	0.96	(0.78 – 1.19)	0.711
	MR RAPS	258	1.13	(0.94 – 1.35)	0.185
	MR Egger slope	258	0.92	(0.57 – 1.48)	0.722
FEV_1/FVC	Outliers filtered:				
	IVW-RE	223	1.15	(1.01 – 1.31)	0.038
	Maximum likelihood	223	1.15	(0.99 – 1.33)	0.062
	Weighted median	223	1.08	(0.87 – 1.34)	0.488
	MR RAPS	223	1.14	(0.98 – 1.32)	0.094
	MR Egger slope	223	1.01	(0.67 – 1.51)	0.972

IVW – RE : Inverse variance weighted – multiplicative random effects
RAPS : Robust adjusted profile score – Huber loss function

Supplementary Table 12: Summary of diagnostic tests conducted for Mendelian Randomization analyses

Phenotype Outcome Pair	MR Egger		Modified Cochran's Q		I^2_{GX}[1]	Mean F	MR Steiger Test[2]		Number of Outliers
	$\beta_{0\ Egger}$	p-value	Q statistic	p-value			Ratio	p-value	
FEV_1 Lung cancer	-0.004	0.53	585.58	2.1×10^{-41}	0.981	52.09	18.24	$<2\times10^{-16}$	36
FEV_1 Adenocarcinoma	0.001	0.83	326.84	3.4×10^{-9}	0.981	53.54	17.01	$<2\times10^{-16}$	23
FEV_1 Squamous	-0.015	0.12	505.33	1.1×10^{-30}	0.981	52.13	11.20	$<2\times10^{-16}$	34
FEV_1/FVC Lung cancer	4.4×10^{-4}	0.90	602.10	1.2×10^{-28}	0.984	63.25	43.25	$<2\times10^{-16}$	38
FEV_1/FVC Adenocarcinoma	-0.003	0.44	419.6	3.4×10^{-9}	0.985	66.11	38.65	$<2\times10^{-16}$	26
FEV_1/FVC Squamous	0.001	0.90	479.9	5.3×10^{-15}	0.984	62.60	28.33	$<2\times10^{-16}$	24
FVC Lung cancer	0.006	0.38	310.4	2.1×10^{-14}	0.979	48.02	13.60	$<2\times10^{-16}$	20
FVC Adenocarcinoma	0.005	0.55	240.2	5.0×10^{-7}	0.979	47.48	9.99	$<2\times10^{-16}$	19
FVC Squamous	0.012	0.30	304.4	2.2×10^{-14}	0.979	48.29	7.62	$<2\times10^{-16}$	18
FEV_1 Never smokers	0.002	0.91	98.54	0.036	0.977	45.03	3.87	3.2×10^{-7}	8
FEV_1/FVC Never smokers	0.014	0.30	142.82	0.023	0.983	59.38	10.58	$<2\times10^{-16}$	9
FVC Never smokers	0.005	0.86	78.07	0.027	0.976	40.82	2.22	4.1×10^{-4}	5
FEV_1 Smokers	-0.005	0.47	419.05	7.7×10^{-17}	0.981	52.02	12.19	$<2\times10^{-16}$	25
FEV_1/FVC Smokers	0.005	0.22	490.72	6.0×10^{-17}	0.984	62.13	27.12	$<2\times10^{-16}$	35
FVC Smokers	0.002	0.82	239.11	2.1×10^{-7}	0.979	48.77	8.76	$<2\times10^{-16}$	
After outlier filtering:									
FEV_1 Lung cancer	0.004	0.36	153.53	0.52	0.980	50.46	21.47	$<2\times10^{-16}$	
FEV_1 Adenocarcinoma	0.004	0.48	153.06	0.79	0.981	52.65	18.77	$<2\times10^{-16}$	
FEV_1 Squamous	-0.004	0.50	141.12	0.80	0.981	52.55	14.09	$<2\times10^{-16}$	
FEV_1/FVC Lung cancer	-0.002	0.44	200.22	0.88	0.984	61.29	47.97	$<2\times10^{-16}$	
FEV_1/FVC Adenocarcinoma	-0.004	0.22	219.37	0.80	0.985	65.64	40.71	$<2\times10^{-16}$	
FEV_1/FVC Squamous	0.002	0.66	228.8	0.64	0.984	61.47	30.67	$<2\times10^{-16}$	
FVC Lung cancer	0.013	0.03	106.2	0.86	0.979	46.83	16.09	$<2\times10^{-16}$	
FVC Adenocarcinoma	0.001	0.93	111.2	0.77	0.978	46.04	10.81	$<2\times10^{-16}$	
FVC Squamous	0.014	0.10	110.4	0.72	0.979	47.99	9.23	$<2\times10^{-16}$	
FEV_1 Never smokers	-0.003	0.89	52.68	0.90	0.978	45.40	4.49	2.4×10^{-12}	
FEV_1/FVC Never smokers	0.001	0.96	81.84	0.93	0.983	59.64	11.56	$<2\times10^{-16}$	
FVC Never smokers	0.001	0.97	45.45	0.73	0.976	40.40	2.45	2.7×10^{-7}	
FEV_1 Smokers	-0.005	0.39	160.75	0.52	0.980	50.70	13.92	$<2\times10^{-16}$	
FEV_1/FVC Smokers	0.002	0.49	181.48	0.98	0.983	59.27	29.55	$<2\times10^{-16}$	
FVC Smokers	0.008	0.24	105.00	0.88	0.979	48.74	10.25	$<2\times10^{-16}$	

1. Values close to 1 indicate that the no measurement error (NOME) assumption is unlikely to be violated. Low I^2_{GX} estimates, less than 0.9, suggest that inferences should be interpreted with caution and adjustment methods should be considered
2. A higher ratio value indicates that the inferred direction is less susceptible to measurement error

Supplementary Table 13: Odds ratio (OR) estimates for a genetically predicted 1-SD decrease in standardized FEV_1 z-scores and 10% decrease in FEV_1/FVC after manual filtering to remove instruments associated with smoking status (ever/never), cigarette pack-years, or bodyfat percentage ($p<1\times10^{-5}$).

MR Estimator	FEV_1 and Lung Cancer							
	Manually filtered				Manual filter and Outlier detection			
	N_{SNPs}	OR	(95% CI)	P-value	N_{SNPs}	OR	(95% CI)	P-value
IVW-RE	168	1.25	(0.98 - 1.59)	0.067	141	1.08	(0.93 - 1.26)	0.320
Maximum likelihood	168	1.25	(1.08 - 1.45)	0.002	141	1.08	(0.93 - 1.27)	0.321
Weighted median	168	1.05	(0.84 - 1.31)	0.661	141	1.04	(0.82 - 1.32)	0.766
MR RAPS	168	1.11	(0.89 - 1.38)	0.364	141	1.05	(0.89 - 1.25)	0.564
Egger intercept	168	-0.011		0.091	141	0.002		0.699
Q statistic	168	484.7		1.0×10^{-32}	141	141.0		0.46
	FEV_1 and Squamous Carcinoma							
	Manually filtered				Manual filter and Outlier detection			
	N_{SNPs}	OR	(95% CI)	P-value	N_{SNPs}	OR	(95% CI)	P-value
IVW-RE	165	2.02	(1.40 - 2.92)	1.9×10^{-4}	137	1.46	(1.16 - 1.84)	0.001
Maximum likelihood	165	2.02	(1.60 - 2.55)	3.1×10^{-9}	137	1.46	(1.14 - 1.87)	0.003
Weighted median	165	1.47	(1.03 - 2.11)	0.035	137	1.36	(0.93 - 2.00)	0.111
MR RAPS	165	1.81	(1.29 - 2.56)	7.1×10^{-4}	137	1.41	(1.09 - 1.83)	0.009
Egger intercept	165	-0.02		0.048	137	-0.007		0.280
MR Egger slope	165	7.56	(1.97 – 29.04)	0.004	137	2.38	(0.95 – 5.94)	0.066
Q statistic	165	435.3		1.9×10^{-26}	137	120.4		0.827
	FEV_1/FVC and Lung Cancer							
	Manually filtered				Manual filter and Outlier detection			
	N_{SNPs}	OR	(95% CI)	P-value	N_{SNPs}	OR	(95% CI)	P-value
IVW-RE	246	1.19	(1.02 - 1.38)	0.03	214	1.09	(0.98 - 1.22)	0.103
Maximum likelihood	246	1.19	(1.07 - 1.32)	0.001	214	1.09	(0.97 - 1.22)	0.133
Weighted median	246	1.11	(0.93 - 1.33)	0.25	214	1.15	(0.96 - 1.39)	0.130
MR RAPS	246	1.12	(0.97 - 1.29)	0.13	214	1.09	(0.96 - 1.22)	0.175
Egger intercept	246	0.001		0.811	214	-0.002		0.444
Q statistic	246	527.1		9.8×10^{-23}	214	188.1		0.890
	FEV_1/FVC and Adenocarcinoma							
	Manually filtered				Manual filter and Outlier detection			
	N_{SNPs}	OR	(95% CI)	P-value	N_{SNPs}	OR	(95% CI)	P-value
IVW-RE	247	1.19	(1.01 - 1.40)	0.043	228	1.24	(1.08 - 1.43)	0.002
Maximum likelihood	247	1.19	(1.04 - 1.36)	0.013	228	1.24	(1.08 - 1.43)	0.002
Weighted median	247	1.34	(1.06 - 1.69)	0.013	228	1.40	(1.10 - 1.77)	0.006
MR RAPS	247	1.20	(1.02 - 1.42)	0.029	228	1.28	(1.10 - 1.48)	0.001
Egger intercept	247	-0.005		0.182	228	-0.006		0.062
Q statistic	247	387.1		3.7×10^{-8}	228	208.5		0.806

IVW – RE : Inverse variance weighted – multiplicative random effects
RAPS : Robust adjusted profile score – Huber loss function

Supplementary Figure 1: Flow chart detailing the main quality control (QC) steps for the genome-wide association analyses of pulmonary phenotypes in the UK Biobank

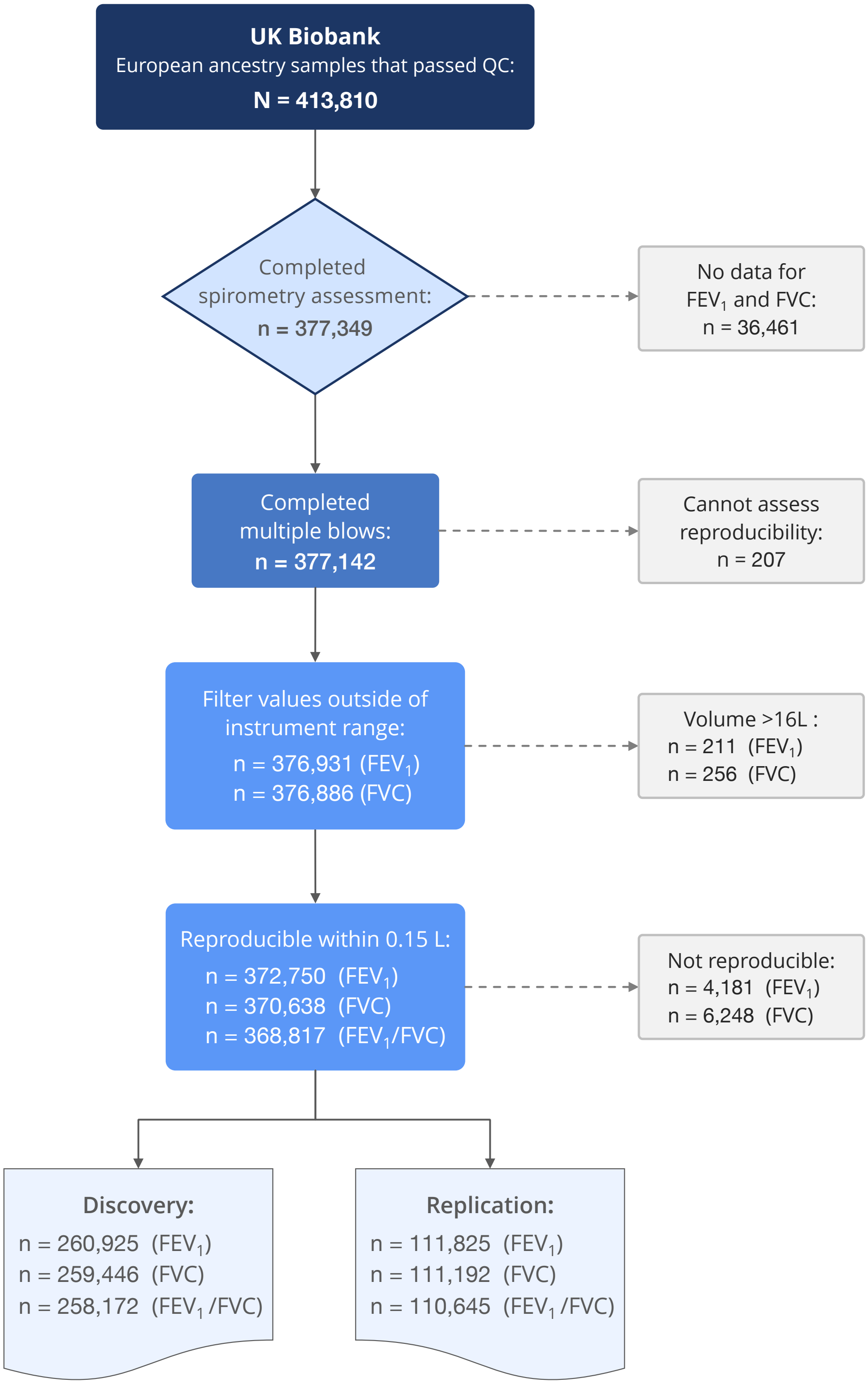

Supplementary Figure 2: Estimated power for Mendelian randomization analyses, based on the available sample size in the OncoArray dataset (29,266 lung cancers, 11,273 adenocarcinoma and 7,426 squamous cell carcinoma cases, and 56,450 controls), and assuming that the genetic instruments explain 5%, 3%, and 2% of variation in FEV_1/FVC, FEV_1, and FVC, respectively.

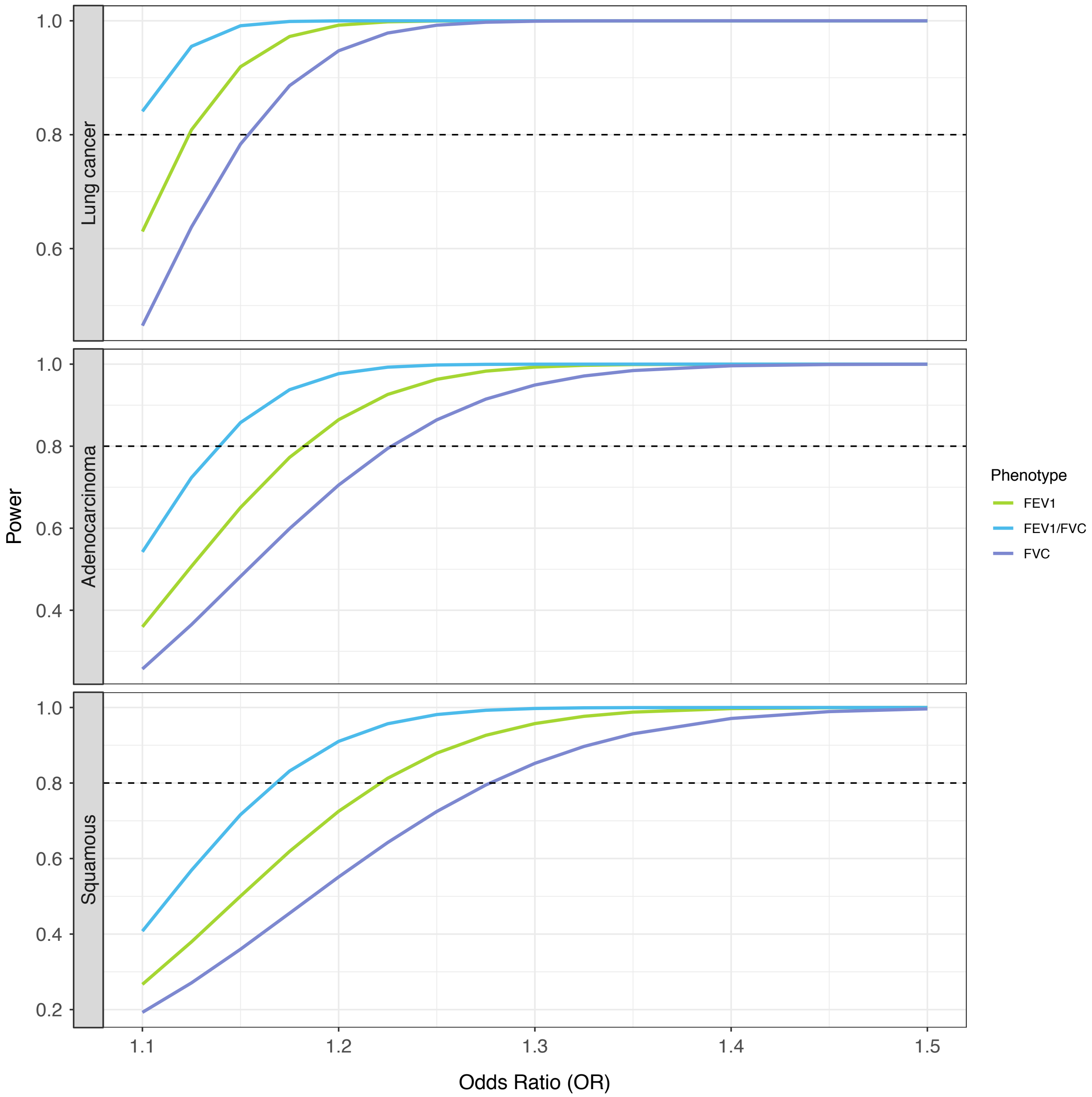

Supplementary Figure 3: Comparison of SNP effect sizes for pulmonary function genetic instruments estimated in the full UK Biobank cohort (original) and in ever smokers only (n=139,562 to 138,019) with adjustment for continuous cigarette pack-years rather than pack-year categories and years since quitting smoking (adjusted). Differences in the effect size distribution were tested using Wilcoxon rank sum test. All correlation coefficients were significant at $p<1\times10^{-40}$.

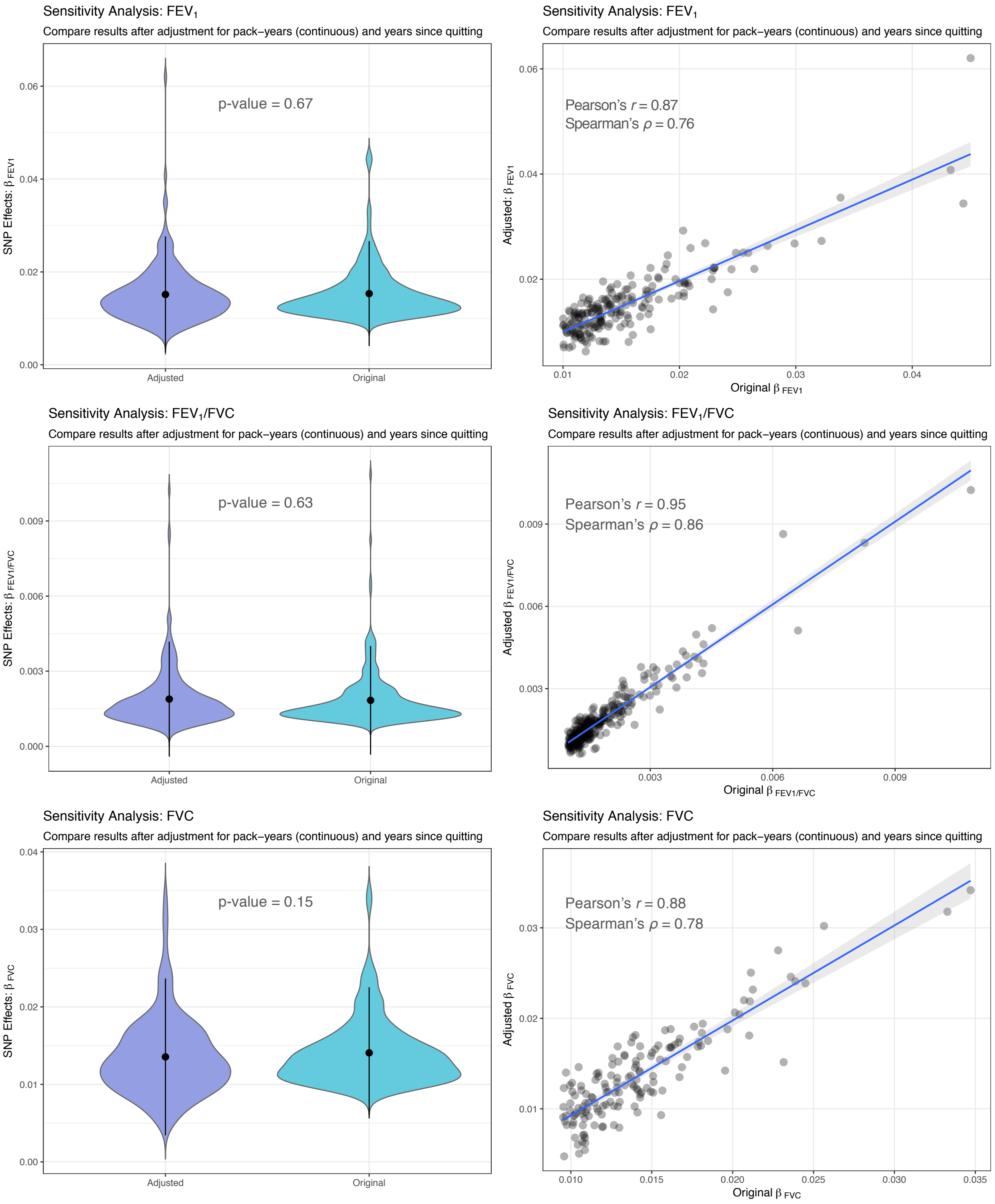

Supplementary Figure 4: Comparison of associations with chronic obstructive pulmonary disease (COPD), defined as $FEV_1/FVC<0.70$) for FEV_1/FVC and FEV_1 genetic instruments. Effect estimates for COPD were estimated in the UK Biobank dataset using logistic regression with adjustment for age, sex, height, cigarette pack-years, genotyping array, and genetic ancestry principal components (PC1-PC15). A total of 54,711 cases and 313,640 controls were included in the analysis. The red solid line corresponds to genome-wide significance ($p=5\times10^{-8}$) and the red dotted line indicates nominal significance (p=0.05).

Associations with COPD: FEV_1/FVC Instruments

COPD cases in UK Biobank based on GOLD criteria: 54,711 cases

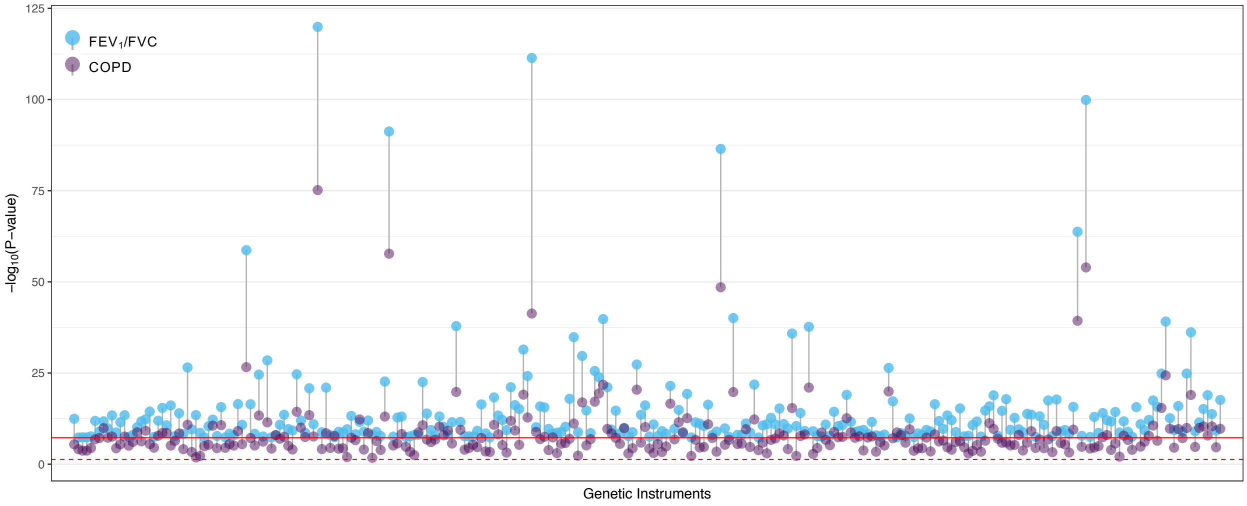

Associations with COPD: FEV_1 Instruments

COPD cases in UK Biobank based on GOLD criteria: 54,711 cases

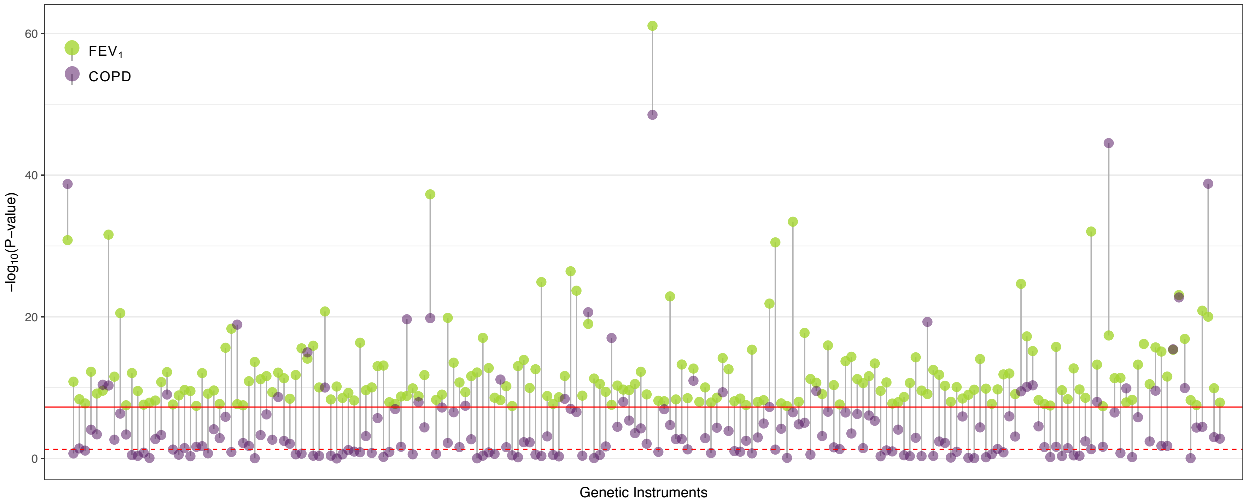

Supplementary Figure 5: Analyses of eQTL effects based on lung tissue gene expression data from the Laval Direction of eQTL effects was considered consistent if increased expression resulted in impaired pulmonary function and increased lung cancer risk (or increased FEV_1 or FEV_1/FVC and an inverse association with lung cancer). Lung cancer associations with $p<5\times10^{-4}$ were considered statistically significant and are labeled in the scatterplot. Odds ratios (OR) for lung cancer per unit increase in predicted gene expression, and corresponding associations with FEV_1 and FEV_1/FVC, are presented for all genes achieving p<0.05 in the lung cancer eQTL Mendelian randomization analysis.

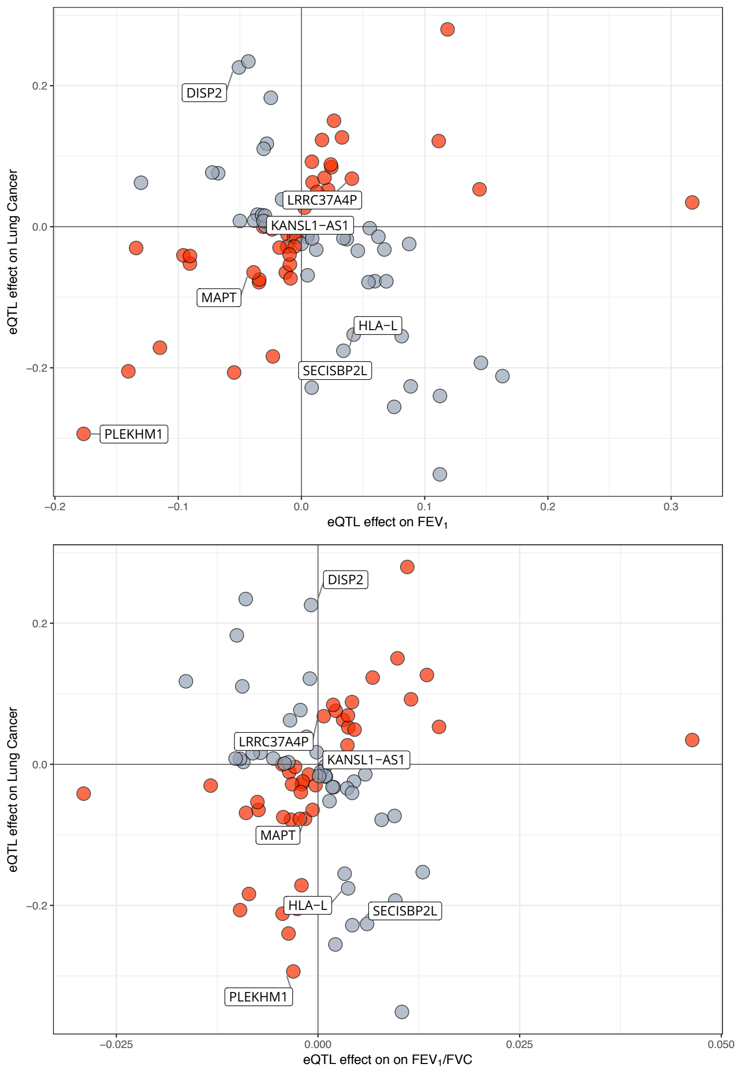

Gene	OR	(95% CI)	$P_{Lung\ cancer}$	P_{FEV_1}	$P_{FEV_1/FVC}$
SECISBP2L	0.80	(0.73-0.86)	5.2×10^{-8}	0.191	3.8×10^{-11}
HLA-L	0.84	(0.78-0.90)	1.6×10^{-6}	4.0×10^{-10}	1.2×10^{-11}
DISP2	1.25	(1.11-1.41)	1.6×10^{-4}	3.9×10^{-8}	0.354
MAPT	0.94	(0.90-0.97)	4.6×10^{-4}	3.0×10^{-62}	4.3×10^{-3}
KANSL1-AS1	0.97	(0.95-0.99)	4.6×10^{-4}	3.0×10^{-62}	4.3×10^{-3}
LRRC37A4P	1.07	(1.03-1.11)	4.6×10^{-4}	3.0×10^{-62}	4.3×10^{-3}
PLEKHM1	0.75	(0.63-0.88)	4.7×10^{-4}	2.9×10^{-62}	4.3×10^{-3}
FBXL19	0.77	(0.65-0.92)	4.4×10^{-3}	2.9×10^{-8}	0.116
AZGP1	1.06	(1.01-1.12)	0.012	0.020	1.3×10^{-15}
TRIM4	0.83	(0.72-0.96)	0.012	4.9×10^{-3}	8.1×10^{-25}
WNT3	0.84	(0.74-0.97)	0.014	9.0×10^{-33}	0.035
RBM6	0.80	(0.66-0.96)	0.016	8.2×10^{-10}	3.1×10^{-5}
HLA-DPA1	1.09	(1.01-1.18)	0.023	7.9×10^{-9}	4.9×10^{-24}
HLA-DPB1	0.81	(0.68-0.97)	0.023	7.9×10^{-9}	4.9×10^{-24}
HSPA4	1.32	(1.04-1.68)	0.023	2.5×10^{-10}	4.9×10^{-9}
UQCR10	1.20	(1.02-1.41)	0.027	0.046	1.4×10^{-15}
HHIPL1	1.26	(1.02-1.56)	0.030	7.9×10^{-3}	4.5×10^{-8}
PDXDC2P	0.98	(0.97-1.00)	0.033	8.3×10^{-25}	0.113
ITGA2	0.92	(0.86-0.99)	0.034	1.6×10^{-10}	3.0×10^{-16}
UQCC1	0.79	(0.63-0.99)	0.039	4.2×10^{-19}	4.1×10^{-3}
MDM4	0.86	(0.74-1.00)	0.044	2.2×10^{-11}	7.1×10^{-3}

Histology-specific effects:

Gene	OR	(95% CI)	P_{Adeno}	Gene	OR	(95% CI)	P_{Squam}
SECISBP2L	0.64	(0.57-0.72)	3.1×10^{-14}	*HLA-L*	0.75	(0.67-0.84)	1.0×10^{-6}
PLEKHM1	0.64	(0.51-0.80)	8.8×10^{-5}	*DISP2*	1.30	(1.08-1.57)	6.2×10^{-3}
LRRC37A4P	1.11	(1.05-1.17)	8.9×10^{-5}	*MDM4*	0.74	(0.58-0.95)	0.017
KANSL1	0.96	(0.93-0.98)	8.9×10^{-5}	*HLA-DRB6*	1.06	(1.01-1.11)	0.022
MAPT	0.91	(0.86-0.95)	8.9×10^{-5}	*STRA13*	0.65	(0.43-0.97)	0.034
WNT3	0.74	(0.61-0.88)	1.1×10^{-3}	*LOC285835*	0.86	(0.74-0.99)	0.039
DISP2	1.21	(1.03-1.42)	0.021	*MARCH3*	0.89	(0.79-1.00)	0.048
MFAP2	0.87	(0.77-0.98)	0.026	*C5orf63*	0.76	(0.58-1.00)	0.048
HLA-L	0.90	(0.81-0.99)	0.034	*SECISBP2L*	1.05	(0.92-1.20)	0.44

Supplementary Figure 6: Visual summary of the Reactome pathway analysis depicting pathways that were significantly over-represented (FDR p<0.05) among genetic instruments for FEV_1/FVC in never smokers. The size of circles (nodes) is proportional to the number of genes included in each pathway and the colors correspond to the q-value for each pathway.

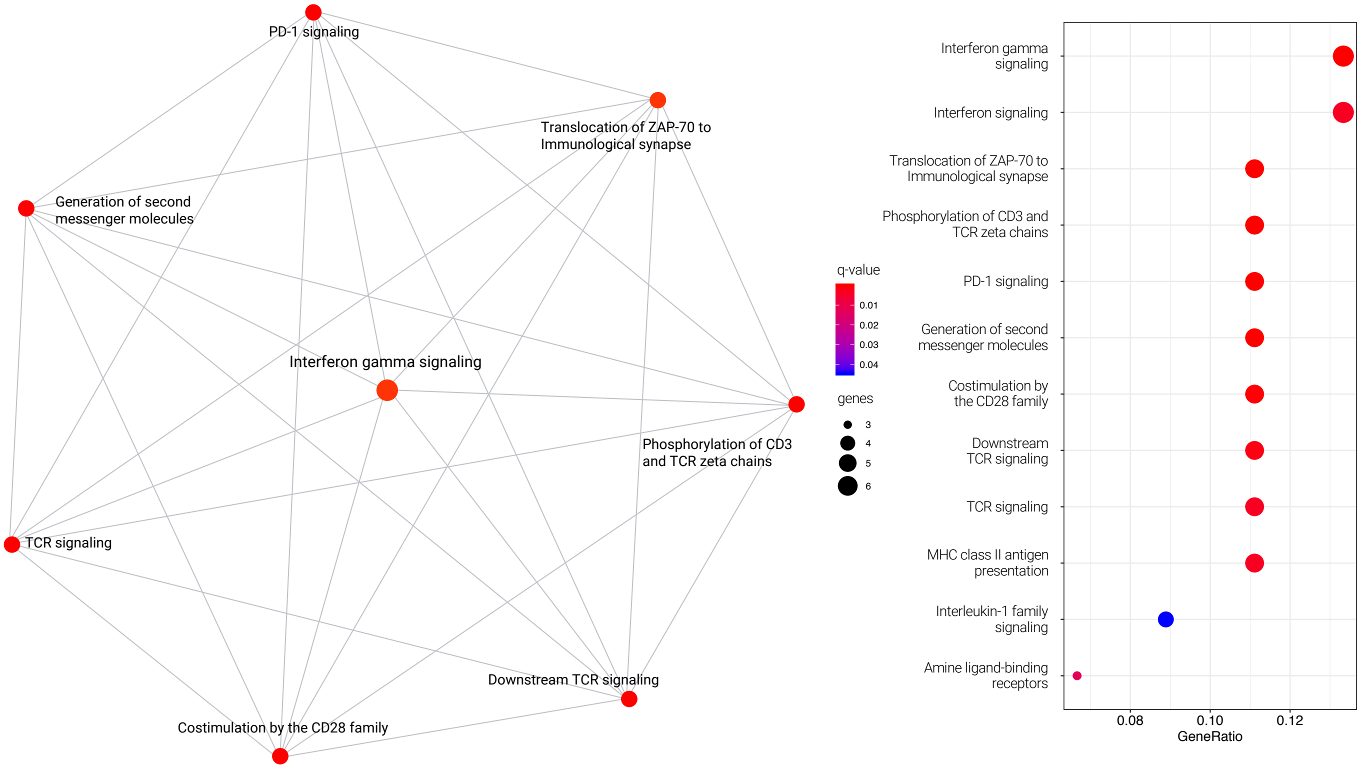

Supplementary Figure 7: Visual summary of the MSigDB pathway analysis depicting pathways that were significantly (FDR p<0.05) among genetic instruments for FEV_1 and FEV_1/FVC.

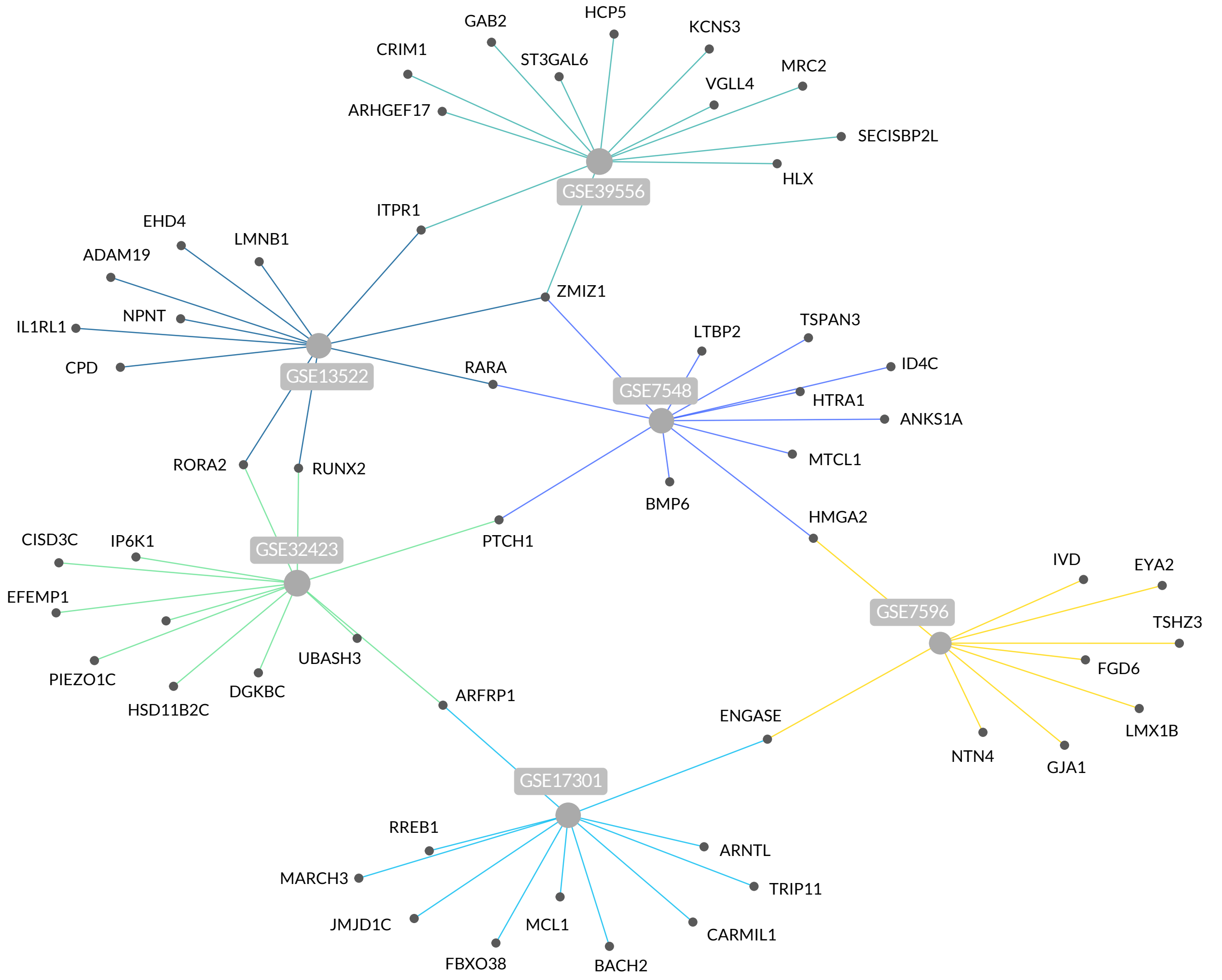

ID	Description	p-value	q-value
GSE32423	Up-regulated in memory CD8 T-cells treated with IL7 vs. IL4 and IL7	1.21×10^{-5}	0.024
GSE39556	Down-regulated in NK cells: untreated vs. pathogen-associated poly(IC)	1.21×10^{-5}	0.024
GSE13522	Down-regulated during intradermal infection: wildtype (BALB/c) vs. IFNAR1 knockout	1.91×10^{-5}	0.024
GSE7596	Down-regulated in T-cells in response to TGF-β: control vs. constantly active AKT1	2.16×10^{-5}	0.024
GSE17301	Up-regulated CD8 T-cells stimulated by INFA2 vs. IFNA5	6.19×10^{-5}	0.045
GSE7548	Up-regulated in lymph node CD4 T-cell: naïve vs. 28 days after immunization	6.19×10^{-5}	0.045
