# Supplementary material for: Immune-mediated genetic pathways resulting in pulmonary function impairment increase lung cancer susceptibility
Source: Nat Commun. 2020 Jan 7;11:27. doi: 10.1038/s41467-019-13855-2 (PMC6946810; doi:10.1038/s41467-019-13855-2)
Supplement: Supplementary file 2 — Description of Additional Supplementary Files [file 41467_2019_13855_MOESM2_ESM.pdf]

Description of Additional Supplementary Files

Supplementary Data 1: Summary statistics for FEV_1 genetic instruments based on a GWAS conducted in the UK Biobank

Supplementary Data 2: Summary statistics for FEV_1/FVC genetic instruments based on a GWAS conducted in the UK Biobank

Supplementary Data 3: Summary statistics for FVC genetic instruments based on a GWAS conducted in the UK Biobank

Supplementary Data 4: Summary statistics for FEV_1 genetic instruments in never smokers based on a GWAS conducted in the UK Biobank

Supplementary Data 5: Summary statistics for FEV_1/FVC genetic instruments in never smokers based on a GWAS conducted in the UK Biobank

Supplementary Data 6: Summary statistics for FVC genetic instruments in never smokers based on a GWAS conducted in the UK Biobank

Supplementary Data 7: Lung expression quantitative trait loci (eQTL) association estimates for FEV_1 and FEV_1/FVC genetic instruments

Supplementary Data 8: Protein quantitative trait loci (pQTL) association estimates for FEV_1 and FEV_1/FVC genetic instruments
